# Viral Etiology of Sudden Sensorineural Hearing Loss: Historical Development and Current Evidence

**DOI:** 10.3390/v18101097

**Published:** 2026-10-03

**Authors:** Shujie Zhao, Xiaozhou Liu, Xinyu Shi, Zhengdong Zhao, Yu Sun

**Affiliations:** 1 Department of Otorhinolaryngology, Union Hospital, Tongji Medical College, Huazhong University of Science and Technology, Wuhan 430022, China; zhaoshujie@hust.edu.cn (S.Z.); 2024xh8023@hust.edu.cn (X.L.); sxy2017@hust.edu.cn (X.S.); d202672628@hust.edu.cn (Z.Z.); 2 Institute of Otorhinolaryngology, Union Hospital, Tongji Medical College, Huazhong University of Science and Technology, Wuhan 430022, China; 3 Hubei Province Clinical Research Center for Deafness and Vertigo, Wuhan 430022, China; 4 Hubei Province Key Laboratory of Oral and Maxillofacial Development and Regeneration, Wuhan 430022, China

**Keywords:** sudden sensorineural hearing loss, viral infection, viral reactivation, inner-ear injury, etiological diagnosis, antiviral therapy

## Abstract

Sudden sensorineural hearing loss (SSNHL) is an otological emergency with complex and heterogeneous etiologies, and most cases remain unexplained and are therefore classified as idiopathic. Viral infection has long been considered an important potential cause of SSNHL; however, the mechanisms underlying virus-associated hearing loss and the causal relationship between viral infection and SSNHL have not been fully elucidated. Current evidence suggests that viruses may induce acute hearing loss through direct invasion of the inner ear, reactivation of latent viruses, immune-mediated injury, and cellular stress and inflammatory responses. This review describes the major viruses associated with SSNHL and their epidemiological and clinical characteristics; summarizes evidence from serological testing, molecular virology, imaging, temporal bone histopathology, and animal studies; and discusses recent advances in the pathogenic mechanisms, etiological diagnosis, and antiviral treatment of virus-associated SSNHL. This review aims to systematically clarify the current evidence and controversies surrounding the viral etiology of SSNHL and to provide a reference for etiological identification, patient stratification, and targeted treatment of virus-associated SSNHL.

## 1. Introduction

Hearing loss is one of the most prevalent sensory impairments worldwide [1]. In 2019, an estimated 1.57 billion people were living with hearing loss globally, including 403.3 million with moderate or more severe disabling hearing impairment; this number is projected to reach 2.45 billion by 2050 [2]. Among the various forms of hearing impairment, sudden sensorineural hearing loss (SSNHL) is an otologic emergency defined as sensorineural hearing loss of at least 30 dB hearing level (dB HL) across three contiguous frequencies occurring suddenly within 72 h [3]. Its annual incidence ranges from approximately 5 to 27 per 100,000 population across regions and it has shown an apparent increase in recent clinical practice [4]. SSNHL is unilateral in the great majority of cases and may involve any frequency range [5]. It is frequently accompanied by tinnitus, vertigo, and aural fullness, and some patients are left with irreversible, permanent hearing loss [6]. Irrespective of age at onset, hearing loss can profoundly affect interpersonal communication, psychosocial well-being, quality of life, and economic independence [7]. Globally, the annual economic cost attributable to hearing loss exceeds US$981 billion, including losses in quality of life, additional healthcare expenditure, productivity losses, and educational support costs [8].

The major clinical challenge in SSNHL is the difficulty in establishing its precise etiology. Even after comprehensive clinical and imaging evaluation, no definite underlying disorder can be identified in more than 70% of cases; these cases are collectively termed idiopathic sudden sensorineural hearing loss (ISSNHL) [6]. In the absence of specific etiologic biomarkers, current clinical practice is often compelled to rely on empirical interventions, including systemic or intratympanic corticosteroids, treatments intended to improve inner-ear microcirculation, and hyperbaric oxygen therapy. This nonspecific, one-size-fits-all approach results in marked interindividual variability in hearing recovery and has limited further improvement in overall treatment efficacy [3,9].

Among the numerous hypotheses proposed to explain the etiology of ISSNHL—including impaired inner-ear microcirculation [10], immune-mediated injury [11,12], rupture of the labyrinthine window membranes [13], and cellular stress responses [14]—the viral etiology hypothesis has long attracted considerable attention because it is supported by a broad range of clinical and pathological observations and is regarded as one of the principal mechanistic explanations for the disorder [5,15]. Epidemiological studies have reported that approximately one-third of patients with SSNHL have a preceding or concurrent acute upper respiratory tract infection [16]. Temporal-bone studies have shown that the inner-ear pathological changes in some patients with SSNHL closely resemble those observed in established viral labyrinthitis [17]. Subsequently, numerous studies have detected evidence of recent viral infection in patients with SSNHL using polymerase chain reaction (PCR) and serological assays [18,19,20], collectively providing important support for the viral hypothesis of SSNHL.

Although an association between viral infection and SSNHL has been widely reported, the overall strength of causal evidence, the feasibility of etiologic diagnosis, and the precise clinical value of targeted antiviral therapy remain controversial and have not been comprehensively synthesized. This review therefore aims to systematically examine the evidence, conclusions, and unresolved questions surrounding the viral etiology of SSNHL, with particular emphasis on: (i) the major viruses closely associated with SSNHL and their epidemiological characteristics; (ii) the pathophysiological and immunological mechanisms by which viral infection may cause SSNHL; and (iii) current approaches to diagnosing virus-associated SSNHL and the clinical evidence supporting antiviral agents as adjunctive therapy. By integrating the available evidence, this review seeks to clarify the etiologic framework for virus-associated SSNHL and to provide a scientific basis for advancing precise, targeted interventions.

### Search Strategy and Selection Criteria

A structured literature search was conducted in PubMed/MEDLINE, Web of Science Core Collection, and Scopus for studies published between January 1950 and April 2026. The core search combined terms related to sudden sensorineural hearing loss (including “sudden sensorineural hearing loss”, “SSNHL”, “sudden hearing loss”, and “sensorineural hearing loss”) with terms related to viral infection (including “virus”, “viral infection”, and “viral reactivation”) and the names of the individual viruses discussed in this review. Additional targeted searches were performed using terms related to etiologic diagnosis, pathogenesis, and antiviral treatment. Reference lists of relevant reviews and key primary studies were also screened to identify additional historical or frequently cited publications. Earlier landmark publications identified through reference-list screening were retained when they were essential to the historical development of the field. During the subsequent revision process, several newly published studies were additionally incorporated to reflect recent advances.

After removal of duplicates, screening for relevance to the scope of this review, and assessment of the full-text content, the eligible literature was further evaluated for inclusion. Ultimately, a total of 211 publications were cited in the final review.

## 2. Clinical and Epidemiological Evidence

### 2.1. Mumps Virus

Mumps virus was among the earliest pathogens implicated by epidemiological evidence in SSNHL. In 1860, Toynbee provided the first description of mumps-associated deafness, noting that hearing loss after mumps was usually sudden and unilateral and could occur without evident abnormalities of the external auditory canal, tympanic membrane, or tympanic cavity [21]. Reports of mumps-associated hearing loss accumulated from the mid-20th century onward, and an important epidemiological feature was its occurrence in infected individuals without typical parotid swelling. In 1988, Nomura et al. tested 53 patients with sudden hearing loss for mumps immunoglobulin M (IgM) and identified three positive cases (5.7%), only one of whom had a history of parotid swelling, suggesting that some cases of sudden hearing loss may be associated with asymptomatic mumps infection [22]. In 1994, Okamoto et al. tested 130 patients and found mumps IgM positivity in nine (6.9%), none of whom had typical manifestations of mumps [23]. Regarding incidence, an office-based prospective study by Hashimoto et al. in 2009 estimated the incidence of mumps-associated deafness at approximately 1 in 1000 infections [24], whereas Takagi et al., using a Japanese insurance claims database, estimated an incidence of approximately 1 in 668 between 2005 and 2017 [25]. Mumps-associated hearing loss is generally severe to profound and responds poorly to conventional corticosteroid therapy [26].

### 2.2. Measles Virus

Measles-associated hearing loss was first reported in 1925, when Shambaugh et al. inferred from medical histories that 8 of 145 children (5.5%) with acquired deafness attending a school for the deaf had hearing loss related to measles [27]. In 1983, Wilson et al. observed measles-virus seroconversion more frequently in patients with ISSNHL than in controls [19]. Measles-associated hearing loss occurs predominantly in children, particularly during critical periods of language development, and may therefore present as severe impairment of speech and language development or deaf-mutism [28]. Before widespread vaccination, the incidence of measles-associated deafness was estimated at approximately 4–9%, typically presenting as bilateral moderate-to-profound sensorineural hearing loss [29]. In low- and middle-income countries and regions with inadequate immunization coverage, measles remains an important cause of severe or profound sensorineural hearing loss in children [30].

### 2.3. Rubella Virus

Hearing loss caused by rubella was first reported in 1943 [31]. Early reports focused mainly on congenital deafness following maternal rubella infection during pregnancy [32,33]. Two large epidemiological investigations attributed 35% and 20.5% of childhood deafness, respectively, to maternal rubella, and the age distributions of affected children closely coincided with the 1940 rubella epidemic in the United Kingdom and the 1943 epidemic in Los Angeles [34,35]. Acquired rubella infection has only rarely been linked to adult SSNHL. In 1980, Osterweil et al. reported unilateral sudden severe sensorineural hearing loss in a 25-year-old woman after rubella infection [36]. In 1994, Kobayashi et al. described a 36-year-old man who developed acute unilateral hearing loss 2 days after a rubella-associated maculopapular rash had subsided [37]. In a paired-serum study of 77 patients with idiopathic sudden hearing loss, Veltri et al. identified rubella-virus antibody seroconversion in five patients (7%) [18]. These findings suggest a possible association between rubella infection and SSNHL, although large cohort or case–control studies are lacking and the true risk remains uncertain.

### 2.4. Herpesviruses

#### 2.4.1. Varicella-Zoster Virus

The association between varicella-zoster virus (VZV) and SSNHL can be traced to Hunt’s 1907 description of herpetic inflammation of the geniculate ganglion accompanied by facial paralysis and audiovestibular symptoms [38]. Herpes zoster oticus and Ramsay Hunt syndrome subsequently became the most representative clinical models of VZV-associated SSNHL. Typical manifestations include unilateral otalgia, vesicles in the external auditory canal or auricle, ipsilateral peripheral facial paralysis, and, in some patients, SSNHL accompanied by tinnitus and vertigo [39,40]. Unlike most cases of ISSNHL, VZV-associated SSNHL often presents with clear evidence of ipsilateral cranial-nerve involvement; however, typical vesicles may be absent, a condition termed zoster sine herpete [39,41]. VZV-associated hearing loss is usually unilateral, commonly affects high frequencies, ranges from severe to profound, and may result in persistent or permanent impairment [42,43]. Epidemiologically, the risk increases with age and immunosuppression, and the disorder is commonly attributable to VZV reactivation [44,45].

#### 2.4.2. Cytomegalovirus

The association between cytomegalovirus (CMV) and hearing loss has been established primarily through studies of congenital infection. The earliest report dates to 1964, when Medearis found deafness in more than 40% of surviving children with cytomegalic inclusion disease [46]. Subsequent studies confirmed congenital CMV infection as a major cause of non-genetic sensorineural hearing loss in children [47,48]. Hearing impairment may occur in infants who are symptomatic at birth as well as in those without evident neonatal manifestations, and it may have delayed onset or follow a fluctuating or progressive course [49,50].

In studies of adult SSNHL, CMV has been included in virological and serological screening panels, with some patients showing CMV-related antibody changes or other evidence of recent infection [19,51]. Most relevant case reports involve immunocompromised populations. CMV labyrinthitis and eighth cranial-nerve involvement presenting with hearing loss have been reported in patients with acquired immunodeficiency syndrome (AIDS) or human immunodeficiency virus (HIV) infection [52,53], and rare cases of CMV otitis media with hearing loss have been described in adult transplant recipients [54]. These reports indicate that, in the setting of severe immunosuppression, CMV may involve the inner ear, middle ear, or auditory-nerve structures.

#### 2.4.3. Herpes Simplex Virus

The proposed relationship between herpes simplex virus (HSV) and SSNHL is based mainly on serological evidence and the hypothesis of reactivation from latent herpesvirus infection. In 1988, Koide et al. found no significant elevation in HSV-1 or HSV-2 antibody titers among patients with sudden hearing loss, whereas the positivity rate for complement-fixing antibodies was significantly increased, suggesting that reactivation of latent HSV may be an important etiological factor [55]. In 2013, Scalia et al. further evaluated HSV-1 immunoglobulin A (IgA) and reported a possible association with sudden hearing loss when titers exceeded 1:80 [56]. Clinically, Rabinstein et al. described a case of bilateral profound SSNHL associated with HSV-1 infection in a patient with oral herpes, subclinical meningitis, and evidence of HSV-1 reactivation; imaging suggested involvement of the eighth cranial nerve and labyrinth [57]. Hearing loss has also been reported after neonatal HSV-2 infection, usually in children with neurological involvement and other severe sequelae [58,59].

#### 2.4.4. Epstein–Barr Virus

In 1964, Gregg and Shaeffer first reported unilateral inner-ear deafness complicating infectious mononucleosis (IM) and proposed that Epstein–Barr virus (EBV) might cause hearing loss [60]. Reports became more detailed after 1980. In 1983, Erzurum et al. described a 16-year-old boy whose initial manifestations of IM were acute cerebellar ataxia and hearing loss; the heterophile-antibody test was positive and the antibody titer against the EBV viral capsid antigen increased fourfold. The ataxia resolved after treatment, whereas the hearing loss persisted [61]. In 1985, Williams et al. reported three young patients who developed sudden, permanent hearing loss 1–4 months after mild EBV infection and demonstrated abnormal cellular immune responses to EBV antigens. They proposed that altered immunoregulation during recovery from primary EBV infection might facilitate viral entry into the inner ear or reactivation of the latent virus [62]. In 2012, Miyashita et al. described a 42-year-old man with EBV-associated aseptic meningitis in whom EBV DNA was detected by PCR in both peripheral blood and cerebrospinal fluid. Right-sided hearing loss, confirmed by pure-tone audiometry, developed approximately 20 days after disease onset; the authors suggested that neuritis of the auditory nerve might have resulted from extension of meningeal inflammation [63].

Small serological and molecular studies have also been conducted in adults with SSNHL. In a 2007 prospective screening study of 48 patients with unilateral ISSNHL, Gross et al. found EBV IgM positivity in three patients (6.25%) [20]. More recently, Lan et al. enrolled 29 adults with idiopathic unilateral SSNHL: serum EBV DNA was positive by quantitative PCR (qPCR) in three patients (10.3%), and viral capsid antigen IgA (VCA-IgA) titers of at least 1:40 were detected in 12 (41.4%). The authors also observed a trend toward an inverse association between EBV DNA load and post-treatment hearing gain [64].

### 2.5. Adenovirus and Other Respiratory Viruses

The hypothesis that respiratory viruses may contribute to SSNHL initially arose from the clinical observation that many patients report a preceding cold-like illness or upper respiratory tract infection. All five patients with SSNHL described by Heller and Lindenberg in 1955 had a history of a mild cold, rhinitis, or nonspecific viral-like illness [65]. In 2021, a large nationwide self-matched case-crossover study found that an acute upper respiratory infection (URI) during the month before SSNHL onset was associated with an increased risk of SSNHL (adjusted odds ratio [aOR], 1.57; 95% confidence interval [CI], 1.20–2.05), and that the association weakened as the exposure window was extended [16]. These studies transformed the early clinical impression of a cold-like prodrome into an epidemiologically testable etiologic clue and suggest that respiratory viral infection may contribute to a subset of SSNHL cases.

Among specific respiratory viruses, adenovirus was one of the earliest pathogens reported in association with SSNHL. In 1967, Jaffe and Maassab studied a patient who developed sudden hearing loss after an upper respiratory infection; adenovirus was isolated from a nasopharyngeal swab, and a fourfold rise in adenovirus-specific antibody titer was documented, suggesting a possible etiologic role [66]. In 2000, García Berrocal et al. performed serological testing in 24 patients with SSNHL and identified evidence of recent infection in three: one was positive for Mycoplasma pneumoniae IgM, one had both M. pneumoniae IgM positivity and an increased complement-fixing antibody titer against influenza A virus, and one showed seroconversion to parainfluenza virus [51]. Following the 2009 H1N1 pandemic, numerous case reports described SSNHL associated with H1N1 infection in adults and children. Hearing loss could be unilateral or bilateral, was often preceded by an influenza-like illness or confirmed by PCR, and could be accompanied by vertigo, vomiting, aural fullness, or tinnitus [67,68,69]. In a 1981 paired-serum virological study of 77 patients with idiopathic sudden hearing loss, Veltri et al. found significant seroconversion to one or more pathogens in 49 patients; influenza B virus accounted for 14 cases, or approximately 18% of the cohort [18].

### 2.6. Human Immunodeficiency Virus

Cases of HIV-associated SSNHL were reported early in the AIDS epidemic. In 1987, Real et al. described the first man with AIDS who developed SSNHL after a series of opportunistic infections [70]. In 1989, Timon and Walsh subsequently reported two HIV-positive patients who presented with SSNHL [71]. Together, these reports established SSNHL as a potential otologic complication of HIV infection.

Hearing impairment in patients with AIDS is heterogeneous and may be conductive, sensorineural, or mixed, as well as unilateral or bilateral. Contributing factors include external- and middle-ear infections, opportunistic infections, meningitis or other central nervous system lesions, exposure to ototoxic drugs, and HIV-related injury to peripheral or central auditory pathways [72,73,74]. Lin et al. summarized previous reports indicating a prevalence of hearing impairment of 29–44% among people with HIV and, in a large cohort study, found that HIV infection was associated with an increased risk of SSNHL among patients aged 18–35 years (adjusted hazard ratio [HR], 2.169; 95% CI, 1.071–4.391) [75].

### 2.7. Lassa Virus

Evidence linking Lassa virus to hearing loss emerged from repeated observations of hearing impairment after Lassa fever in endemic regions of West Africa. In 1972, White first reported deafness after laboratory-confirmed Lassa virus infection, with an incidence of approximately 17.4% (4/23) [76]. Subsequent studies suggested that, on average, 33.2% (range, 4–75%) of Lassa fever survivors developed SSNHL [77,78]. Given an estimated 500,000 cases of Lassa fever annually in West Africa, this complication constitutes a substantial public-health burden [77].

Lassa virus-associated sensorineural hearing loss has most commonly been described during convalescence, although onset during the acute phase has also been reported [79,80]. Epidemiological studies further indicate that the risk of sensorineural hearing loss is higher among patients with Lassa fever than among patients with other febrile illnesses. In a 1990 prospective audiological assessment of 69 hospitalized febrile patients, Cummins et al. found sensorineural hearing loss in 14 of 49 patients (29%) with confirmed Lassa fever and in none of 20 febrile controls [79]. In a similar study, Ibekwe et al. found early-onset bilateral sensorineural hearing loss in 5 of 37 patients (13.5%) with Lassa fever confirmed by reverse transcription PCR (RT-PCR), compared with 0% of febrile controls [81].

### 2.8. Hepatitis B Virus

Case reports of hepatitis B virus (HBV)-associated SSNHL are uncommon. The most representative report was published by Huang et al. in 2009 and involved a patient with chronic HBV carriage who developed sudden left-sided SSNHL and persistent high-pitched tinnitus [82]. Abnormal liver function, elevated HBV DNA, and imaging evidence of labyrinthitis suggested that an acute exacerbation of chronic hepatitis B might have precipitated the hearing loss [82]. Because HBV infection is highly prevalent, its potential association with SSNHL has also been investigated in cross-sectional audiological studies and population-based databases. In a 2017 retrospective cohort study using the Taiwan Longitudinal Health Insurance Database, Chen et al. found a significantly higher incidence of SSNHL in patients with viral hepatitis than in controls (0.38% vs. 0.19%); within the HBV subgroup, the adjusted HR for SSNHL was 5.110 (95% CI, 4.495–5.809) [83]. In 2018, Tsai et al. conducted a dedicated HBV cohort study and likewise found that HBV infection was associated with an increased risk of SSNHL, with an adjusted HR of 1.315 (95% CI, 1.148–1.506) [84]. Nasab et al. compared 95 patients with HBV infection and 97 healthy controls and found significantly higher mean hearing thresholds in the HBV group at 500, 1000, and 2000 Hz [85].

Available clinical studies suggest that HBV-associated SSNHL is commonly unilateral and may be accompanied by tinnitus, but not necessarily by vertigo, jaundice, or overt systemic symptoms of infection [82,86]. HBV may also affect the auditory system indirectly through extrahepatic immune vasculitic mechanisms, such as polyarteritis nodosa [87,88].

### 2.9. Enteroviruses

A possible association between enterovirus infection and SSNHL was first reported in 2003. A previously healthy young man developed sudden severe bilateral sensorineural hearing loss and tinnitus; enterovirus PCR was positive in cerebrospinal fluid, and echovirus type 4 of the same serotype was subsequently cultured from stool. Hearing improved substantially within 5–10 days after methylprednisolone pulse therapy [89]. Existing epidemiological evidence derives mainly from viral-screening studies in patients with SSNHL, but results vary markedly across studies. In 2004, Mentel et al. tested 55 patients with SSNHL and detected enteroviral RNA by RT-PCR in 40% of patients and in none of the controls [90]. By contrast, in the 2007 prospective screening study by Gross et al., only one of 48 patients with unilateral SSNHL was positive by enterovirus RT-PCR, corresponding to a rate of 2.08% [20].

### 2.10. West Nile Virus

SSNHL remains a rare complication of West Nile virus (WNV) infection. In 2006, McBride et al. first reported a woman with WNV meningoencephalitis and severe bilateral sensorineural hearing loss. Treatment included piperacillin/tazobactam, vancomycin, prednisone, azathioprine, acetaminophen, and supportive care, and her hearing ultimately improved [91]. In 2019, Pradhan et al. reported three patients with WNV encephalitis who developed behavioral and cognitive disturbances together with sudden, irreversible, bilateral symmetric sensorineural deafness [92]. In contrast to typical adult SSNHL, which is usually isolated and unilateral, reported WNV-associated cases are often bilateral and may be accompanied by tremor, headache, altered consciousness or behavior, acute flaccid paralysis, quadriplegia, oculomotor palsy, disequilibrium, positional vertigo, or gait abnormalities. Hearing outcomes have varied considerably [92,93,94,95,96].

Regarding frequency, long-term follow-up of the Houston West Nile Cohort showed that, among 60 individuals with WNV infection, 20 had hearing loss on short-term neurological evaluation, while four had gait instability and eight had tremor [97]. In a 1-year follow-up study after an outbreak of WNV encephalitis in Kerala, India, 27 of 30 survivors underwent clinical assessment; hearing loss, impaired smell, and dizziness were each reported in 7.4%, suggesting that auditory and balance disturbances may persist as long-term neurological sequelae of WNV encephalitis [98].

### 2.11. Dengue Virus

Dengue virus was first reported as a possible cause of SSNHL in 2015. A 60-year-old man developed bilateral otitis media with effusion and sensorineural hearing loss 5 days after the onset of dengue hemorrhagic fever, and hearing had not recovered after 7 months of follow-up [99]. Collectively, available cases suggest that dengue-associated SSNHL generally develops during classical or severe dengue and is often accompanied by systemic manifestations such as thrombocytopenia, bleeding tendency, neurological involvement, or microcirculatory disturbance. Associated otologic symptoms may include tinnitus, vertigo, hyperacusis, aural fullness, or disequilibrium. Onset typically occurs several days after fever or after severe complications emerge, while the severity and prognosis of hearing loss vary substantially [99,100,101,102,103].

Preliminary epidemiological studies indicate that abnormal hearing thresholds may occur in patients with dengue, although sample sizes remain small. In a 2021 prospective pilot study of 10 patients with confirmed dengue, Soni et al. found that two patients reported hearing loss after fever and were confirmed to have mild bilateral sensorineural hearing loss; a third patient had no subjective complaint but showed bilateral high-frequency hearing loss [104]. A subsequent case–control study in 2026 enrolled 40 patients with dengue confirmed by nonstructural protein 1 (NS1) antigen and 39 controls with other febrile illnesses. Hearing loss occurred in 15.2% of the dengue group and in none of the controls (odds ratio [OR], 34.6; 95% CI, 1.97–610), and both air- and bone-conduction thresholds were significantly elevated in the dengue group [105].

### 2.12. Zika Virus

The earliest reports of SSNHL after Zika virus (ZIKV) infection emerged during the 2015 outbreak in Bahia, Brazil. In 2017, Vinhaes et al. reported three adults who developed hearing loss after an acute rash illness during the outbreak; one had laboratory-confirmed ZIKV infection, whereas the other two also had elevated dengue-virus IgM. Hearing recovered completely or partially within 1 month in all three patients [106]. Martins et al. subsequently reported two patients with ZIKV infection. One developed unilateral moderate sensorineural hearing loss, whereas the other had normal bilateral pure-tone thresholds but abnormal contralateral acoustic reflexes and abnormalities in selected otoacoustic-emission and brainstem auditory evoked-potential measurements, suggesting possible neural auditory-pathway involvement. However, both patients were positive for both ZIKV and dengue virus (DENV) immunoglobulin G (IgG), precluding definitive attribution of the auditory findings to ZIKV [107]. The World Health Organization (WHO) has emphasized that a positive ZIKV IgM result must be interpreted in light of cross-reactivity with related flaviviruses, particularly dengue virus [108]. Accordingly, in regions where dengue and ZIKV co-circulate, a single positive IgM or IgG result is insufficient to establish ZIKV as the cause of SSNHL. Attribution is more convincing when sudden hearing loss has a clear temporal relationship to an acute ZIKV-like rash illness, mosquito exposure, or residence in an endemic area and is supported by RT-PCR, acute-phase IgM, a dynamic increase in plaque reduction neutralization test (PRNT) titers, or evidence excluding dengue infection [109].

### 2.13. Severe Acute Respiratory Syndrome Coronavirus 2

Since the onset of the coronavirus disease 2019 (COVID-19) pandemic, numerous studies have examined the relationship between severe acute respiratory syndrome coronavirus 2 (SARS-CoV-2) and SSNHL. Early case reports showed that COVID-19-associated SSNHL could develop after severe pneumonia or intensive care unit (ICU) treatment, but also in patients with mild disease or almost no respiratory symptoms. In April 2020, Sriwijitalai and Wiwanitkit first reported sensorineural hearing loss in a patient with COVID-19 in Thailand [110]. Degen et al. subsequently described a previously healthy 60-year-old man who was admitted to the ICU with COVID-19 pneumonia and later developed bilateral severe hearing loss and tinnitus. Magnetic resonance imaging (MRI) showed enhancement of the right cochlea, partial loss of fluid signal in the basal turn of the right cochlea, and meningeal enhancement along the basal right temporal lobe; the authors proposed that viral meningitis had extended to involve the cochlea [111]. In July 2020, Abdel Rhman and Abdel Wahid reported an asymptomatic SARS-CoV-2-positive patient who presented with progressively worsening tinnitus and sudden left-sided SSNHL. Pure-tone audiometry demonstrated severe left-sided sensorineural hearing loss, suggesting that SSNHL may be the presenting manifestation of SARS-CoV-2 infection in some patients [112]. Overall, published cases have most often described acute unilateral SSNHL accompanied by tinnitus, vertigo, or dizziness, with frequent high-frequency involvement and variable severity. Most patients received conventional treatment with oral, intravenous, or intratympanic corticosteroids, sometimes combined with hyperbaric oxygen therapy. Outcomes were heterogeneous: partial recovery was most frequently reported, whereas approximately one-quarter of cases showed no meaningful improvement or persistent hearing loss [113,114,115,116].

Subsequent population-based studies provided epidemiological evidence for an association between SARS-CoV-2 infection and SSNHL. In a 2024 nationwide Korean cohort of 6,716,879 adults aged 20–39 years without pre-existing hearing loss, Kim et al. reported an SSNHL incidence of 1.8 per 10,000 person-months in the COVID-19 group compared with 0.5 per 10,000 person-months in the non-COVID-19 group; in the fully adjusted model, the adjusted subdistribution HR was 3.52 (95% CI, 3.23–3.83) [117]. In a 2025 case–control study of adults who underwent pure-tone audiometry at 32 government hospitals in Malaysia, Abdul Rahim et al. included 187 patients with SSNHL and 935 controls. Previous COVID-19 infection was present in 16 of 187 patients with SSNHL (8.6%) and 44 of 935 controls (4.7%), corresponding to an adjusted OR of 2.49 (95% CI, 1.176–5.260) [118].

The hypothesis that viral infection may cause SSNHL was initially supported mainly by case reports, case series, epidemiological observations, and serological surveys. Overall, the strength of evidence differs considerably among viruses, and systemic evidence of viral infection cannot be identified in all patients with ISSNHL, indicating that viral infection probably accounts for only a subset of cases rather than representing a single unifying cause. These virus-specific differences in clinical presentation, diagnostic evidence, therapeutic response, and strength of causal evidence are summarized in Table 1. Because SSNHL has an abrupt onset, direct sampling of the inner ear is difficult, and patients may present after the optimal diagnostic window, evidence for most implicated viruses is derived from retrospective case–control studies and case series. The scarcity of high-quality, large prospective cohorts means that much of the evidence remains associative rather than demonstrably causal. Nevertheless, despite these methodological limitations, epidemiological studies have provided an important foundation for investigating the viral hypothesis and have offered limited evidence supporting empirical antiviral therapy in selected clinical contexts.

## 3. Diagnosis

### 3.1. Confirmation of SSNHL and Exclusion of Alternative Diagnoses

The 2019 AAO-HNSF guideline recommends obtaining audiometry as soon as possible and within 14 days of symptom onset to confirm SSNHL [3]. Initial assessment should distinguish sensorineural from conductive hearing loss and use the history and physical examination to identify bilateral or recurrent hearing loss and focal neurological findings that may indicate a specific cause [154,155]. Tympanometry helps exclude middle-ear disease, whereas otoacoustic emissions, auditory brainstem response (ABR), electrocochleography, and vestibular testing—including the video head impulse test and electronystagmography—may further localize the lesion and determine whether labyrinthine or retrocochlear structures are involved [3,156,157]. MRI of the internal auditory canal is the preferred imaging modality because retrocochlear lesions, particularly vestibular schwannoma, must be excluded [128]. Accordingly, guidelines recommend MRI or ABR for the evaluation of retrocochlear pathology and advise against routine head computed tomography (CT) during the initial assessment of sudden hearing loss [3].

### 3.2. Clinical Features and Exposure History Suggesting a Viral Etiology

The diagnosis of virus-associated SSNHL should begin with characteristic clinical manifestations and a targeted history rather than indiscriminate viral-panel testing in all patients with sudden hearing loss. A viral etiology is more credible when SSNHL is concordant with a characteristic infectious syndrome, a documented exposure, a biologically plausible time window, and objective pathogen-specific evidence [3,5,6]. Attribution is substantially stronger when SSNHL accompanies a distinctive infectious syndrome than when it is supported only by a recent nonspecific upper respiratory illness or a single positive antibody result. For example, when SSNHL occurs together with otalgia, vesicles on the auricle or in the external auditory canal, peripheral facial paralysis, taste disturbance, tinnitus, and vertigo, VZV-associated Ramsay Hunt syndrome or herpes zoster oticus should be considered first [39,158]. By contrast, sudden hearing loss accompanied by parotid or other salivary-gland swelling, contact with mumps, or an outbreak in a school or other congregate setting should prompt consideration of mumps virus [26].

Exposure history should systematically address travel and residence, mosquito exposure, contact with animals or rodents, congregate living, vaccination status, immunosuppression, sexual exposure, and recently confirmed infections [5,6,159]. When a relevant exposure is identified, interpretation should incorporate the pathogen’s incubation period, the diagnostic window, and the temporal relationship between infection and hearing loss. The suspected pathogen and the timing of presentation should therefore guide the choice of specimen and pathogen-specific test.

### 3.3. Pathogen-Specific Testing: Specimen Selection and Timing

#### 3.3.1. Serological Testing

Because direct sampling of the inner ear is generally not feasible, clinical diagnosis usually relies on indirect serological evidence. IgM positivity may suggest a recent primary infection, whereas reactivation of a previous infection may be assessed through dynamic changes in immunoglobulin G (IgG) in paired sera. However, serological interpretation is more complex for herpesviruses because prior infection and persistent IgG positivity are common, while reactivation may occur without a diagnostic IgM response or a characteristic change in IgG titer [15,129].

Virus-specific serological markers may have diagnostic value in selected settings. Mumps IgM, for example, can identify subclinical or inapparent mumps infection. In 2001, Fukuda et al. reported mumps IgM positivity in 7.2% of patients with SSNHL [119], although this proportion has declined with widespread vaccination and updated assay methods. Using a second-generation IgM enzyme immunoassay (EIA-IgM) kit, a later study found anti-mumps IgM positivity in only 1.0% of patients with SSNHL [120]. For EBV, IgA responses to the early antigen (EA) and viral capsid antigen (VCA) may be measured by indirect hemagglutination [64]. Positive SARS-CoV-2 IgG has also been detected several weeks after SSNHL, suggesting that infection may have coincided with the otologic symptoms [160].

Serological diagnosis nevertheless has major limitations. Most importantly, previous infection with common herpesviruses is highly prevalent in adults, and serological findings therefore require cautious interpretation [161]. Moreover, endogenous reactivation often does not elicit an IgM response, potentially leading to underestimation of the contribution of viral reactivation to SSNHL [129]. Some studies have also found no increase in sensitive markers of systemic viral infection in patients with sudden hearing loss. Pitkäranta and Julkunen, for example, detected neither interferon production nor interferon-induced gene expression in peripheral blood samples from patients with SSNHL, supporting the view that SSNHL is generally not accompanied by systemic viral infection [162].

#### 3.3.2. Nucleic Acid Testing

Molecular methods such as PCR directly detect viral nucleic acids in clinical specimens and therefore provide relatively specific evidence of viral presence or infection [163]. However, PCR positivity alone does not establish active viral replication, particularly for herpesviruses, because latent viral DNA may persist in tissues. PCR results should therefore be interpreted in the context of specimen type, timing of sampling, viral biology, and clinical presentation. Potential specimens include peripheral blood, nasopharyngeal swabs, perilymph, and cerebrospinal fluid, with specimen selection guided by the suspected pathogen and stage of infection. Real-time PCR of serum or peripheral blood mononuclear cells (PBMCs) from patients with severe bilateral sensorineural hearing loss has detected DNA from several herpesviruses [131]. Serum EBV DNA can also be quantified by real-time PCR; in a study of 29 patients with SSNHL, Lan et al. found EBV positivity by qPCR in three patients (10.3%), and higher viral PCR titers were associated with poorer recovery of hearing thresholds among corticosteroid-treated patients [64].

Detection of viral nucleic acids in perilymph provides the most direct evidence obtainable near the inner ear. However, because perilymph sampling is invasive, most studies have collected specimens during procedures such as cochlear implantation. In a prospective study, Sugiura et al. performed real-time PCR on perilymph and PBMCs from cochlear implant recipients and detected CMV DNA in the perilymph of two patients with congenital CMV infection [131]. Another study similarly detected human CMV DNA by real-time PCR in perilymph from patients with sensorineural hearing loss or deafness [132]. Notably, Di Nardo et al. identified CMV and HSV-1 genomes in perilymph from patients with sensorineural hearing loss who had no serological or clinical history of congenital infection. These findings support the hypothesis that herpesviruses may establish latency in the spiral ganglion after postnatal infection and later damage the cochlea through reactivation, while also highlighting a possible dissociation between peripheral-blood findings and the actual virological status of the inner ear [133]. In children with idiopathic sensorineural hearing loss, combined perilymph PCR and serology identified evidence of CMV or HSV infection in approximately 16.7% of cases [134]. Nevertheless, because of the potential harm associated with obtaining inner-ear specimens in living patients, a direct causal relationship between acute viral infection and SSNHL has not been conclusively established [157]. These limitations have stimulated growing interest in blood-based liquid-biopsy approaches, including circulating nucleic acids and extracellular vesicles, which may provide indirect molecular information on inner-ear injury without requiring direct sampling of the inner ear; these approaches are discussed in detail in Section 3.4.3.

#### 3.3.3. Cerebrospinal Fluid Testing

When SSNHL is suspected to coexist with central nervous system involvement, such as meningitis, encephalitis, or rhombencephalitis, lumbar puncture and cerebrospinal fluid (CSF) analysis may provide important etiologic information. A representative case involved VZV rhombencephalitis presenting initially as SSNHL: the patient had only unilateral sudden hearing loss, but MRI of the internal auditory canal unexpectedly revealed posterior fossa inflammation consistent with rhombencephalitis. Lumbar puncture confirmed VZV encephalitis, and the patient received intravenous acyclovir. A herpetiform rash appeared only several weeks later, while follow-up MRI showed near-complete resolution of the lesion [128]. This case illustrates that CSF PCR and antibody testing may be crucial for identifying a viral cause when SSNHL is accompanied by neurological clues. In HSV-associated sudden hearing loss, HSV-1 DNA has likewise been detected in CSF in association with enhancement of the eighth cranial nerve [57].

### 3.4. Nonspecific Supportive and Prognostic Markers

#### 3.4.1. 3D-FLAIR MRI and Quantitative Imaging

Three-dimensional fluid-attenuated inversion recovery (3D-FLAIR) imaging is highly sensitive to subtle inner-ear abnormalities. In 2006, Sugiura et al. first reported precontrast 3D-FLAIR hyperintensity in the affected inner ear of approximately half of patients with ISSNHL and proposed that this finding might reflect microhemorrhage, increased vascular permeability with protein extravasation, or destruction of inner-ear cells [164]. Compared with T1- and T2-weighted imaging, 3D-FLAIR is more sensitive to signal abnormalities caused by elevated protein concentrations and can demonstrate disruption of the blood–labyrinth barrier as enhancement within the inner-ear fluid spaces [165]. It can therefore assist in detecting abnormalities within the labyrinth itself, although such findings are not specific for a viral etiology.

Beyond conventional visual assessment, quantitative imaging and radiomic analysis may further increase the information obtainable from inner-ear MRI. A systematic review and meta-analysis involving 638 patients found abnormal high signal intensity on 3D-FLAIR imaging in approximately 29% of patients with sudden hearing loss and showed that these abnormalities were associated with more severe initial hearing loss and poorer final hearing outcomes [166]. More recent prospective work has similarly demonstrated that incorporation of 3D-FLAIR findings into clinical and audiological assessment can improve prognostic stratification [167]. Radiomics extends this approach by extracting high-dimensional intensity, texture, and spatial features that may capture subtle tissue alterations not readily appreciable by visual inspection. The feasibility of MRI-based radiomic analysis has been demonstrated in another inner-ear disorder, Ménière disease [168], whereas radiomic studies specifically focused on SSNHL remain limited and have not yet established a validated 3D-FLAIR-based model. Importantly, no radiomic signature has been shown to distinguish virus-associated SSNHL from other etiologic subtypes. Future studies will require standardized imaging protocols, reproducible inner-ear segmentation, multicenter external validation, and integration of imaging features with molecular and virological biomarkers before radiomics can contribute to etiologic diagnosis.

#### 3.4.2. Peripheral Blood Inflammatory Markers

Because inflammation is thought to participate in the pathogenesis of both viral and immune-mediated SSNHL, inexpensive and readily available peripheral blood inflammatory markers have attracted interest. The neutrophil-to-lymphocyte ratio (NLR) can be calculated from a routine complete blood count, is rapidly available, and incurs virtually no additional cost [169]. Multiple studies have shown that both the NLR and platelet-to-lymphocyte ratio (PLR) are significantly higher in patients with SSNHL than in controls and that the NLR is inversely associated with the extent of hearing recovery, suggesting potential diagnostic and prognostic value [170].

These markers are not specific for a viral etiology, however, and their methodological validity remains debated. Some authors have argued that use of the NLR alone, without accompanying measures such as C-reactive protein, erythrocyte sedimentation rate, tumor necrosis factor alpha (TNF-α), or interleukin 6 (IL-6), is insufficient to establish the presence of inflammation and may not accurately predict prognosis [171]. Inflammatory markers are therefore more appropriately used as supportive indicators of inflammatory or immune activity and as prognostic aids rather than as confirmatory evidence of viral infection.

#### 3.4.3. Emerging Non-Invasive Molecular Biomarkers

Circulating microRNAs (miRNAs) have emerged as candidate non-invasive biomarkers for SSNHL because their expression in peripheral blood may reflect pathological processes occurring at otherwise inaccessible sites. In a study of 21 patients with idiopathic SSNHL and 24 healthy controls, five circulating miRNAs showed significantly altered expression, and a combined panel of miR-183, miR-210, miR-18b, and miR-23a distinguished patients from controls with a sensitivity of 80.95% and a specificity of 87.50%; several miRNA levels were also associated with hearing status and treatment outcome [172]. More recently, altered expression of miR-210-3p, miR-23a-3p, and miR-18b-5p was reproduced in an independent North American cohort, providing preliminary evidence that some circulating miRNA changes may be detectable across different populations [173]. Extracellular-vesicle-associated miRNAs may offer additional information because vesicular cargo can be isolated from peripheral blood. Serum exosomal profiling in patients with SSNHL identified 18 differentially expressed miRNAs, including increased miR-93-3p [174], while subsequent profiling of plasma exosomes from patients with profound SSNHL identified distinct miRNA patterns associated with hearing recovery [175]. These findings support the potential use of circulating and exosomal miRNAs for disease detection and prognostic stratification; however, the studies remain small, and none of the identified miRNA signatures has been validated as specific for a viral etiology.

Liquid-biopsy approaches based on cell-free DNA (cfDNA) and circulating extracellular vesicles provide another potential route for non-invasive molecular assessment. Whole-genome sequencing of plasma cfDNA from 84 patients with SSNHL identified distinct sequence-coverage patterns between patients with and without hearing recovery, with NOTCH1, APOE, and RELB emerging as independent prognostic factors [176]. Extracellular-vesicle profiling has likewise revealed candidate biomarkers: plasma EV-derived complement C3 has been associated with hearing-loss severity and early treatment response in profound SSNHL [177], whereas proteomic analysis of circulating exosomes has identified differential expression of proteins including RPS2, RPL19, ACO2, and APOE [178]. Of particular interest, sensory-hair-cell-derived exosomes expressing myosin VIIa have been isolated from human perilymph, providing proof of principle that extracellular vesicles can carry molecular information from specific inner-ear cell populations [179]. Although the latter approach currently requires invasive perilymph sampling, future detection of inner-ear-cell-derived vesicles in peripheral blood could provide a more direct molecular window into cochlear pathology. However, these liquid-biopsy approaches remain exploratory and lack specificity for a viral etiology.

In summary, no single test can currently establish in a living patient that SSNHL was caused by viral infection. Diagnosis of virus-associated SSNHL should therefore follow a staged, integrated approach: pure-tone audiometry and appropriate audiological and imaging examinations should first confirm SSNHL and exclude conductive or retrocochlear disease; characteristic infectious manifestations, exposure history, and a biologically plausible temporal relationship should then raise suspicion of a viral etiology; pathogen-specific testing should be selected according to the suspected virus, appropriate specimen, and diagnostic time window; and nonspecific imaging, inflammatory, and emerging molecular biomarkers may provide supportive or prognostic information. Future studies integrating non-invasive molecular biomarkers with pathogen-specific testing and advanced imaging may provide a more reliable framework for distinguishing virus-associated SSNHL from other etiologic subtypes.

## 4. Mechanisms

### 4.1. Direct Viral Invasion

Direct viral invasion of the inner ear depends first on the route by which a virus reaches it. Chen et al. summarized two proposed routes for mumps virus: hematogenous spread to the inner ear during viremia and entry from the CSF into the perilymphatic space through the cochlear aqueduct or internal auditory canal [129]. Animal experiments have identified a third route, namely entry from the middle ear through the cochlear windows [180]. Temporal-bone histopathology provided the earliest evidence for direct invasion. By the 1950s, characteristic lesions had been identified in temporal bones from patients with virus-associated deafness. Deafness related to mumps, measles, and congenital rubella showed degenerative changes predominantly involving the cochleosaccular structures [121,123,125], whereas CMV infection involved the endolymphatic system and cochlear and vestibular structures and was accompanied by inclusion-bearing cells [135]. Studies of measles labyrinthitis further distinguished two pathological patterns: one in which the internal auditory canal served as the route of entry and degeneration predominated in spiral ganglion cells, and another in which the stria vascularis appeared to be the portal of viral entry and degenerative changes affected multiple structures within the endolymphatic compartment [124]. Importantly, temporal-bone changes in ISSNHL closely resemble those observed in established viral labyrinthitis. The absence of new bone formation and of cochlear lesions suggestive of vascular occlusion, together with preservation of the vascular architecture, has therefore been interpreted as favoring a viral rather than a vascular etiology in at least some specimens [181].

A series of classic animal experiments subsequently provided direct experimental support for virus-mediated inner-ear injury. After inoculating HSV directly into the scala tympani of guinea pigs, Nomura et al. observed several morphological abnormalities of the tectorial membrane, including atrophy, curling, and punctate projections; immunofluorescence and electron microscopy confirmed that these changes were attributable to HSV infection. Notably, identical tectorial-membrane abnormalities were observed in both temporal bones of a patient with SSNHL, and HSV antigen was detected in the morphologically normal, uninoculated contralateral cochlea in the animal model, suggesting viral dissemination within the inner ear [143]. Stokroos et al. subsequently established an HSV-1 inner-ear infection model through the round-window route. Inoculated animals rapidly developed sensorineural hearing loss together with loss of outer hair cells, atrophy of the stria vascularis and tectorial membrane, fibrosis of the perilymphatic spaces, and widespread viral antigen within the cochlea [144]. Studies of Lassa virus further supported hematogenous invasion: after intraperitoneal inoculation, surviving animals developed hearing loss late in infection or during early recovery, with injury primarily affecting spiral ganglion neurons and highly vascularized cochlear cells. Lassa-virus antigen was detected in damaged regions, indicating that the virus reached the inner ear through the circulation [129,148]. Using human inner-ear tissue and inner-ear organoids derived from human induced pluripotent stem cells, Jeong et al. showed that hair cells, Schwann-cell precursors, and otic prosensory cells express viral-entry factors including angiotensin-converting enzyme 2 (ACE2), transmembrane serine protease 2 (TMPRSS2), and furin (FURIN); organoid hair cells could serve as targets of SARS-CoV-2 infection [152].

Recent studies have begun to delineate the molecular pathways by which direct viral invasion leads to cell death in the inner ear [182]. In 2021, Hayashi et al. found that cochlear hair cells were protected against viral infection by surrounding supporting cells and cells of Kölliker’s organ; however, infected supporting cells induced hair-cell death by producing tumor necrosis factor-related apoptosis-inducing ligand (TRAIL). Hair-cell death did not exhibit typical caspase-3-dependent apoptosis and was suppressed by necroptosis inhibitors such as necrostatin-1 and ponatinib, suggesting that TRAIL-mediated necroptosis may be an important mechanism of virus-associated hair-cell injury [183]. Thus, even when hair cells are not themselves infected, infection of neighboring cells may induce their death through paracrine signaling. In 2026, Liu et al. identified a distinct pathway in intranasally infected K18-ACE2 mice: SARS-CoV-2 directly entered the inner ear and selectively targeted spiral ganglion neurons (SGNs). The viral spike protein inhibited mechanistic target of rapamycin (mTOR) signaling, promoted abnormal aggregation of Ras-GTPase-activating protein-binding protein 1 (G3BP1)-positive stress granules, drove neuronal phase separation, and ultimately increased apoptosis [153].

### 4.2. Reactivation of Latent Virus

Reactivation from latency is considered a central mechanism by which herpesviruses may cause SSNHL [184]. Early molecular pathological studies detected HSV-1 DNA in the human spiral ganglion [140]. Subsequently, HSV-1-specific DNA and the latency-associated transcript (LAT) were identified in the trigeminal, geniculate, vestibular, and spiral ganglia, suggesting that sensory ganglia of the head and neck may form a continuous network of herpesvirus latency [141,142]. In 2015, Liu et al. established an in vitro model of latent infection in spiral ganglion cells and showed that HSV-1 could enter an LAT-positive latent state without release of infectious virus, then resume lytic replication and produce infectious virions after chemical stimulation [145]. Although these studies do not prove that clinical SSNHL is caused by HSV reactivation, they establish the biological plausibility of this mechanism.

Ramsay Hunt syndrome provides a prototypical example of viral reactivation. After primary infection, VZV remains latent in the geniculate, vestibular, and spiral ganglia and may reactivate when host immunity declines, causing facial paralysis and sudden hearing loss [129]. PCR detection of the VZV genome in auricular and oral vesicles, the facial-nerve sheath, middle-ear mucus, and CSF has led investigators to propose that VZV spreads from the facial nerve to the vestibulocochlear nerve through anastomosing neural branches or reaches the inner ear through the oval and round windows [42]. Clinical serology also supports reactivation. In one patient with bilateral SSNHL and oral herpes but no previous history of febrile herpes, acute-phase anti-HSV-1 IgM was not substantially elevated, whereas IgG was markedly increased, suggesting reactivation of a pre-existing latent HSV-1 infection [57,129]. The high seroprevalence of herpesviruses in the general population further suggests that HSV-, VZV-, or EBV-associated SSNHL in adults is theoretically more likely to result from reactivation of latent infection than from primary infection [185].

### 4.3. Immune-Mediated Injury

An increasing body of evidence suggests that immune-mediated mechanisms contribute importantly to virus-associated SSNHL [186]. Classic antigen-challenge experiments demonstrated that, although the inner ear is protected by the blood–labyrinth barrier, it is not completely immunologically isolated. Antigen entry into the inner ear can elicit both local and systemic immune responses, and the endolymphatic sac has an important role in inner-ear antigen processing, antibody production, and cellular infiltration [187,188]. Hashimoto et al. showed that systemic activation of innate immunity can enhance adaptive immune responses to a local cochlear antigen, as reflected by leukocyte infiltration and increased interleukin-1 beta (IL-1β) expression [189]. Thus, after viral infection, tissue injury, or inflammatory stimulation, even small amounts of inner-ear antigen may be recognized by the immune system and provoke an amplified response in susceptible individuals.

Molecular mimicry is a classic mechanism proposed to explain autoimmune inner-ear injury after infection. Viral infection may induce autoimmunity in two principal ways. First, structural similarity between viral antigens and inner-ear self-antigens may produce cross-reactive antibodies or T cells. Second, viral inflammation may disrupt inner-ear barriers or damage local tissue, exposing previously sequestered antigens and thereby triggering an adaptive immune response [190,191]. Under this model, the virus need not persist within the inner ear or directly infect hair cells; its primary role may be to initiate or amplify an aberrant immune response against inner-ear structures. Viruses may also injure the inner ear indirectly by inducing immunothrombotic abnormalities or vasculitis-like reactions. In 2024, Chen et al. proposed that immune responses triggered by viral peptides may induce antiphospholipid antibodies, which can rise transiently during the acute phase of SSNHL and may impair inner-ear perfusion through transient microthrombosis or endothelial injury [192]. In a cynomolgus macaque model, Cashman et al. observed rapidly developing sensorineural hearing loss after Lassa-virus exposure and perivascular inflammation near the cochlear nerve, with pathology resembling immune-mediated systemic vasculitis [149]. In 2022, Maruyama et al. further showed in mice that depletion of CD4+ T cells prevented hearing loss, suggesting that CD4+ T-cell-mediated immunity may initiate or amplify injury to neurovascular structures of the inner ear [150].

Although these mechanisms overlap in vivo, the immune-mediated processes discussed above primarily emphasize antigen-driven adaptive immune responses and immune-related vascular injury, whereas the following section focuses on local innate immune sensing, cellular stress, and subsequent inflammatory cascade amplification within the cochlea.

### 4.4. Cellular Stress and Inflammatory Cascades

The cellular-stress hypothesis proposes that viral infection may contribute to SSNHL by activating local innate immune and stress responses in the inner ear. The cochlea contains macrophages, supporting cells, and spiral-ligament fibrocytes that participate in immune recognition and inflammatory amplification [193,194,195,196]. When viral nucleic acids, viral proteins, or damage-associated molecular patterns released after infection are sensed by pattern-recognition receptors, they can activate expression of interferon-related genes, chemokines, adhesion molecules, and proinflammatory cytokines, thereby recruiting peripheral immune cells into the inner ear and establishing a local inflammatory microenvironment [193,197].

Reactive oxygen species (ROS) and inflammasomes are among the best-studied downstream mediators of virus-associated hearing loss. Although murine cytomegalovirus (MCMV) is distinct from human cytomegalovirus (HCMV), experimental MCMV models provide indirect mechanistic evidence regarding virus-associated cochlear injury. MCMV infection increases ROS levels in the cochlea and in cultured spiral ganglion neurons and activates the nucleotide-binding oligomerization domain-like receptor family pyrin domain-containing 3 (NLRP3) inflammasome, leading to caspase-1 activation and maturation and release of IL-1β and interleukin-18 (IL-18) [198]. More recent work has linked inflammasome activation to programmed cell death. Li et al. found that MCMV-induced spiral ganglion neuron death involved both pyroptosis and apoptosis, predominantly through coactivation of caspase-1 and caspase-8. Blocking assembly of the apoptosis-associated speck-like protein containing a caspase recruitment domain (ASC) inflammasome inhibited these pathways, reduced neuronal death, and improved hearing [199]. Another study showed that MCMV uses the mixed-lineage kinase (MLK) family to regulate p53/c-Jun N-terminal kinase (JNK)-mediated apoptosis and NLRP3/caspase-1-mediated pyroptosis; the MLK inhibitor URMC-099 attenuated virus-associated spiral ganglion injury and hearing loss [200]. These findings suggest that virus-associated hearing loss may arise from an amplifying network that integrates oxidative stress, inflammasome activation, pyroptosis, apoptosis, and neuroinflammation.

Inflammatory cascades not only damage neurosensory structures but may also exacerbate hearing loss by disrupting cochlear microcirculation. TNF-α is an important molecular link between inflammation and regulation of inner-ear blood flow. Animal experiments have shown that TNF-α enhances sphingosine-1-phosphate (S1P) signaling, shifts cochlear microvessels toward a proconstrictive state, and reduces cochlear blood flow [201]. Further studies found that both the TNF inhibitor etanercept and the S1P receptor 2 antagonist JTE-013 reversed TNF-induced reductions in cochlear blood flow [202]. Masuda et al. proposed that severe ISSNHL may involve simultaneous activation of several nuclear factor kappa B (NF-κB) pathways: reduced natural killer (NK)-cell activity, acute neutrophilia, and elevated IL-6 may collectively promote aberrant NF-κB activation in the cochlear lateral wall, thereby establishing a positive-feedback loop of cellular stress and inflammatory amplification [203].

The intensity, anatomical distribution, and duration of cellular stress and inflammatory cascades may determine whether hearing injury is reversible. In 2025, Smith et al. found in an MCMV model that early cochlear viral load correlated with expression of inflammatory mediators including interferon-induced protein with tetratricopeptide repeats 1 (IFIT1), TNF-α, and interferon gamma (IFN-γ), as well as with cochlear pathology and subsequent hearing loss. Early administration of an anti-MCMV monoclonal antibody reduced viral load, attenuated cochlear inflammation, limited spiral ganglion injury, and prevented sensorineural hearing loss [204].

Taken together, the mechanisms described above are unlikely to operate independently in vivo. Primary viral infection or reactivation of latent virus may initiate direct infection of inner-ear cells and/or antigen exposure, thereby activating local innate and adaptive immune responses and promoting the release of proinflammatory cytokines and chemokines. These responses may be further amplified by oxidative stress and NLRP3 inflammasome activation, while inflammatory mediators such as TNF-α can enhance S1P signaling, promote cochlear microvascular constriction, and reduce inner-ear blood flow. These overlapping inflammatory, vascular, and cellular-stress pathways ultimately converge on injury to hair cells, spiral ganglion neurons, and the stria vascularis, with apoptosis, necroptosis, and pyroptosis contributing to neurosensory damage. The relative contribution of each pathway is likely to vary according to the virus, host immune response, and stage of infection, but together they provide an integrated framework linking primary infection or viral reactivation to acute hearing loss.

## 5. Treatment

The viral hypothesis of SSNHL has suggested a new therapeutic rationale: if sudden hearing loss in a subset of patients is virus-related, suppressing viral replication within an early therapeutic window might reduce cochlear and auditory-nerve injury [204]. Acyclovir, which inhibits replication of HSV and VZV, was among the earliest antiviral agents investigated for SSNHL [137]. Several studies reported favorable hearing recovery after acyclovir. In a retrospective analysis of 102 patients with SSNHL, Yamaguchi et al. found an overall response rate—defined as complete, marked, or partial recovery—of 84.0% among 25 patients treated with acyclovir, compared with 61.0% among those who did not receive acyclovir; the difference was statistically significant [136]. Scalia et al. reported an 81% response rate to acyclovir monotherapy among patients with high HSV-1 IgA titers [56]. Animal studies yielded similar findings. In a guinea-pig model of HSV-1 labyrinthitis, Stokroos et al. found that combined prednisolone and acyclovir produced earlier hearing recovery and less cochlear destruction than prednisolone alone [137].

Beginning in the late 1990s, antiviral therapy for SSNHL was evaluated in randomized controlled trials (RCTs). In the earliest prospective randomized double-blind trial, Stokroos et al. compared prednisolone plus intravenous acyclovir with prednisolone plus placebo and found no statistically significant additional benefit from acyclovir [138]. A Cochrane review of four randomized trials involving 257 participants reached a similar conclusion: adding antiviral therapy to corticosteroids did not provide consistent benefit [139].

However, most trials enrolled patients with ISSNHL without adequate etiologic stratification, whereas virus-associated SSNHL may account for only a minority of cases. Analyses of an unselected population may therefore underestimate treatment effects in a true viral subgroup. A further consideration is whether antiviral agents can achieve effective concentrations at sites of inner-ear infection. Systemically administered drugs must cross the blood–labyrinth barrier (BLB), a tightly regulated vascular interface that restricts many circulating compounds from entering the cochlea [205]. Insufficient or variable drug exposure within the inner ear may therefore reduce the effectiveness of systemic antiviral therapy. Intratympanic administration provides an alternative route that can largely bypass the BLB, allowing drugs placed in the middle ear to enter the cochlea mainly through the round window membrane (RWM) and, to a lesser extent, the oval window [206,207]. However, local delivery is also subject to substantial pharmacokinetic variability, including interindividual differences in RWM permeability, drug clearance through the Eustachian tube, and the formation of a pronounced basal-to-apical concentration gradient as drugs diffuse within the cochlea [206]. Sustained-release systems such as hydrogels can prolong drug contact with the RWM and increase local retention [208]. Experimental studies have demonstrated the feasibility of intratympanic delivery of antiviral agents such as ganciclovir and cidofovir, including hydrogel-based formulations; however, some regimens have also produced transient or frequency-specific elevations in hearing thresholds [209].

Antiviral therapy should also be selected according to the specific pathogen. For HSV- or VZV-associated disease, acyclovir or valacyclovir has a clearer etiologic rationale when a characteristic clinical syndrome or virological evidence is present, particularly when treatment is initiated early; however, evidence for hearing-specific benefit remains less robust than that for recovery of other neurological manifestations [126,139]. For CMV-associated hearing loss, a randomized trial in children with symptomatic congenital CMV disease involving the central nervous system showed that 6 weeks of intravenous ganciclovir reduced subsequent hearing deterioration, although treatment was associated with a high rate of neutropenia [210]. A subsequent randomized trial comparing 6 months with 6 weeks of oral valganciclovir found no significant additional benefit in the primary short-term best-ear hearing outcome at 6 months, but extended treatment was associated with better total-ear hearing outcomes at 12 and 24 months [130]. These findings support pathogen-directed antiviral therapy in a well-defined CMV disease context but should not be extrapolated directly to adult CMV-associated SSNHL.

More recently, early administration of neutralizing anti-CMV monoclonal antibodies in a neonatal murine CMV model reduced cochlear viral load, inflammation, histopathological injury, and subsequent sensorineural hearing loss, suggesting that early control of viral burden may interrupt subsequent disease progression [204]. However, this monoclonal-antibody strategy remains preclinical and has not yet been validated in human SSNHL. Overall, current evidence does not support routine antiviral therapy for etiologically unselected patients with SSNHL. Treatment efficacy may depend on several interrelated factors, including identification of the causative pathogen, whether active viral replication is still present, the timing of treatment initiation, actual drug exposure within the inner ear, and whether cochlear injury has already become irreversible at presentation. Antiviral therapy is therefore biologically more plausible in patients with a well-supported viral etiology when treatment can be administered within an appropriate therapeutic window. Future clinical trials should incorporate etiologic stratification and pathogen-specific treatment while also considering inner-ear pharmacokinetics, BLB permeability, and, where appropriate, optimized local-delivery strategies. Emerging approaches, including intratympanic antiviral delivery and neutralizing monoclonal antibodies, remain investigational and require further evaluation of efficacy, dosing, and potential ototoxicity before routine clinical application. Accordingly, based on the available evidence, the 2019 AAO-HNSF guideline recommends against the routine use of antiviral therapy for SSNHL [3].

## 6. Future Directions

Taken together, evidence from clinical cases, temporal-bone pathology, animal models, and molecular virological studies provides converging evidence that viral infection may contribute to inner-ear injury and acute hearing loss, supporting its role as a potential etiologic factor in a subset of SSNHL cases. The greatest barrier to clinical translation of the viral hypothesis, however, remains diagnosis. Evidence of peripheral infection does not necessarily reflect the virological status of the inner ear, whereas perilymph, spiral ganglia, and cochlear tissue cannot be obtained safely and routinely from most patients with sudden hearing loss. Peripheral inflammatory and coagulation indices, cytokines, and metabolic biomarkers offer potential new directions for etiologic diagnosis, but most remain at the stage of association studies and have not yet been integrated into a reliable diagnostic framework. Inadequate etiologic stratification also directly constrains both antiviral trials and clinical practice [211]. In hearing loss with definitive virological evidence, such as congenital CMV infection and Ramsay Hunt syndrome, antiviral therapy has a clearer etiologic rationale and some evidence of clinical benefit [126,130]. Future therapeutic research should therefore move beyond asking whether all patients with SSNHL require antivirals and instead determine which virus-associated subtypes are most likely to benefit, within what therapeutic window, and from which antiviral or combined antiviral–anti-inflammatory strategy.

## Figures and Tables

**Table 1:** Summary and grading of clinical, diagnostic, therapeutic, and causal evidence for viral-associated SSNHL.

Virus	Primary Clinical Phenotype Relevant to SSNHL	Diagnostic Markers/Clues	Therapeutic Response Pattern	Major Causal Evidence	Evidence Level *	Strength of Association with SSNHL †/Key Limitation
Mumps virus	Typically sudden unilateral severe-to-profound SNHL; may occur without parotid swelling.	Mumps IgM; paired serology; parotid/salivary-gland swelling, contact or outbreak history. Subclinical infection is possible [22,23,119,120].	Generally poor response to conventional corticosteroid therapy [26]; no established SSNHL-specific antiviral regimen.	Mumps IgM was detected in 3/53 (5.7%) patients with sudden hearing loss; no non-SSNHL comparison group was included [22].	4	Strong. Repeated clinical and serological observations, consistent epidemiological evidence, and compatible human temporal-bone pathology support the association. Importantly, direct isolation of mumps virus from perilymph after sudden hearing loss provides highly specific virological evidence of inner-ear infection, substantially strengthening the causal link.
				Mumps IgM was detected in 9/130 tested patients (6.9%) with sudden hearing loss, none with typical mumps manifestations; no non-SSNHL comparison group was included [23].	4	
				Mumps seroconversion was significantly more frequent in patients with idiopathic sudden hearing loss than in controls [19].	3b	
				A prospective office-based surveillance study followed 7400 clinically diagnosed mumps patients and identified seven confirmed hearing-loss cases (approximately 1 in 1000); no non-mumps comparison group was included [24].	4	
				A retrospective claims-based incidence study identified 102 mumps-deafness cases among 68,112 mumps patients (approximately 1 in 668), without a non-mumps comparison group [25].	4	
				Human temporal-bone pathology demonstrated cochleosaccular degeneration in mumps-associated deafness [121].	4	
				Mumps virus was directly isolated by culture from perilymph obtained from a patient who developed complete sudden hearing loss within two days of mumps onset [122].	4	
Measles virus	Evidence predominantly concerns bilateral moderate-to-profound acquired childhood SNHL, rather than classical adult SSNHL.	Clinical measles syndrome and exposure/vaccination history; virus-specific serology and seroconversion.	No virus-specific SSNHL treatment-response pattern established in the reviewed evidence.	Measles-virus seroconversion was significantly more frequent in patients with idiopathic sudden hearing loss than in controls [19].	3b	Moderate. Human pathological and comparative serological evidence support biological plausibility, but much of the clinical literature concerns childhood or nonsudden SNHL rather than strictly defined SSNHL.
				Descriptive clinical/epidemiological studies linked measles infection to acquired childhood deafness (non-SSNHL phenotypes) [27,28,30].	4 (indirect evidence)	
				Human temporal-bone studies demonstrated measles-associated inner-ear pathology, including cochleosaccular injury and characteristic labyrinthitis patterns [123,124].	4	
Rubella virus	Predominantly associated with congenital SNHL; acquired adult SSNHL is rare and usually unilateral.	Rubella-compatible rash or recent illness; paired serology demonstrating recent infection or seroconversion.	No established treatment pattern. Hearing improvement after corticosteroid therapy has been reported in an isolated adult case of acquired rubella-associated SNHL [37], but the evidence is insufficient to infer treatment efficacy.	Two adult case reports described acute unilateral severe or sudden hearing loss temporally related to rubella infection [36,37].	4	Limited. Strong evidence exists for congenital rubella hearing loss, but direct evidence for acquired SSNHL remains sparse.
				Rubella antibody seroconversion was identified in 5/77 (7%) patients with idiopathic sudden hearing loss in an uncontrolled paired-serum series [18].	4	
				Historical population-level observations linked maternal rubella epidemics with childhood deafness; this ecological evidence supports cochlear tropism but is not direct SSNHL evidence [34,35].	2c (indirect evidence)	
				Human temporal-bone pathology demonstrated inner-ear injury following maternal rubella [125].	4 (indirect evidence)	
Varicella-zoster virus (VZV)	Usually unilateral high-frequency, severe-to-profound SSNHL in herpes zoster oticus/Ramsay Hunt syndrome; tinnitus, vertigo, otalgia, auricular/EAC vesicles and facial palsy are characteristic. Zoster sine herpete may occur.	Characteristic vesicles and facial palsy; VZV PCR or antibody testing in appropriate specimens; CSF PCR/antibody testing when neurological involvement is suspected; MRI may reveal associated neural/CNS inflammation.	Early acyclovir plus corticosteroid therapy in Ramsay Hunt syndrome has a clear etiologic rationale and some evidence of clinical benefit [126]; this should not be extrapolated to unselected ISSNHL.	Clinical observational series consistently describe hearing loss as part of herpes zoster oticus/Ramsay Hunt syndrome [38,39,40,41,42,43].	4	Strong. Ramsay Hunt syndrome represents one of the clearest virus-specific clinical models of SSNHL, with characteristic phenotype, comparative serological evidence and pathogen-specific molecular support.
				VZV seroconversion was significantly more frequent in patients with idiopathic sudden hearing loss than in controls [19].	3b	
				VZV DNA was detected by PCR in auricular/oral vesicles, the facial nerve sheath, middle-ear mucosa, and cerebrospinal fluid of patients with Ramsay Hunt syndrome, whereas corresponding samples from control patients with facial palsy of other etiologies were negative [127].	3b	
				A case of SSNHL as the presenting manifestation of VZV rhombencephalitis was supported by CSF investigation and characteristic neuroimaging [128].	4	
				Evidence that VZV can remain latent in cranial sensory ganglia and reactivate provides mechanism-based support for auditory injury [129].	5	
Cytomegalovirus (CMV)	Strongly associated with congenital, delayed-onset, fluctuating or progressive SNHL; adult SSNHL is uncommon and occurs particularly in immunocompromised patients.	CMV serology and blood PCR according to clinical context; perilymph PCR provides direct inner-ear evidence but is not routinely feasible.	Valganciclovir has clinical evidence in symptomatic congenital CMV disease [130], but not in typical adult SSNHL.	CMV seroconversion was significantly more frequent in patients with idiopathic sudden hearing loss than in controls [19].	3b	Limited for adult SSNHL. Comparative serological and direct inner-ear evidence exists, but most mechanistic and pathological evidence derives from congenital or other non-SSNHL phenotypes.
				Prospective/cross-sectional patient series detected CMV DNA in human perilymph or endolabyrinthic fluid, but lacked an appropriate non-SNHL comparison group [131,132,133,134].	4	
				A human temporal-bone case study demonstrated CMV inclusion-bearing cells and inner-ear involvement [135].	4	
Herpes simplex virus (HSV)	Adult HSV-associated SSNHL may be unilateral or bilateral and severe/profound.	HSV-specific serological findings should be interpreted cautiously; PCR detection in blood or CSF, and in rare cases perilymph, cannot by itself determine whether the virus is actively replicating and should therefore be interpreted in conjunction with the clinical presentation.	Retrospective/subgroup studies suggested benefit from acyclovir [56,136], and HSV-labyrinthitis animals benefited from acyclovir plus prednisolone [137]; however, randomized trials and a Cochrane review in unselected ISSNHL showed no consistent additional benefit [138,139].	Controlled serological studies found that the positivity rate for complement-fixing antibodies was significantly higher in patients with sudden hearing loss [55].	3b	Limited. Although serological findings, isolated virologically supported cases, and experimental models suggest a possible role for HSV, direct human evidence linking HSV reactivation to SSNHL remains sparse and inconsistent.
				Human tissue studies detected HSV-1 DNA and/or latency-associated transcripts in spiral and other cranial sensory ganglia [140,141,142].	4 (indirect evidence)	
				HSV-1 genomes were sought in human endolabyrinthic fluid in a small profoundly deaf patient series [133].	4 (indirect evidence)	
				Guinea-pig HSV inner-ear infection models reproduced SNHL, outer-hair-cell loss, strial and tectorial-membrane injury, and cochlear viral antigen [143,144].	5	
				An in-vitro spiral-ganglion model demonstrated HSV-1 latency followed by experimentally induced reactivation and productive viral replication [145].	5 (indirect evidence)	
Epstein–Barr virus (EBV)	Sudden or persistent SNHL may occur during or after infectious mononucleosis or EBV-associated meningitis; unilateral ISSNHL has also been reported.	EBV VCA/EA antibodies and serum qPCR; blood and CSF PCR are particularly informative when CNS disease is present.	Higher EBV DNA load showed a trend toward poorer post-treatment hearing gain [64]; no established EBV-specific antiviral treatment benefit.	Individual cases reported sudden hearing loss during or after infectious mononucleosis, including cases with supportive serological changes [60,61,62].	4	Limited. Pathogen-specific findings have been reported in individual cases and small SSNHL patient series, but no robust controlled human study directly demonstrates an association between EBV infection and SSNHL.
				EBV DNA was detected by PCR in peripheral blood and CSF in a patient with EBV-associated meningitis followed by unilateral hearing loss [63].	4	
				A prospective screening series of 48 unilateral ISSNHL patients found EBV IgM positivity in 3 (6.25%); no non-SSNHL comparison group was included [20].	4	
				A 29-patient unilateral ISSNHL series detected serum EBV DNA by qPCR in 3 (10.3%) and VCA-IgA ≥ 1:40 in 12 (41.4%); no separate non-SSNHL control group was enrolled [64].	4	
Adenovirus and other respiratory viruses	SSNHL may follow a cold-like or influenza-like illness; influenza-associated cases may be unilateral or bilateral and accompanied by tinnitus, vertigo, vomiting or aural fullness.	Respiratory-virus PCR from appropriate respiratory specimens; paired virus-specific serology when useful. A nonspecific recent URI alone does not establish viral causation.	Treatment responses are heterogeneous. Rapid recovery after high-dose corticosteroids has been reported in an isolated H1N1-associated SSNHL case [67], whereas profound bilateral deafness has occurred despite oseltamivir treatment [68]; no virus-specific therapy has been shown to improve hearing outcomes.	A nationwide self-matched case-crossover study found that acute URI during the month before SSNHL onset was associated with increased SSNHL risk (aOR 1.57, 95% CI 1.20–2.05) [16].	3b	Limited. Recent respiratory infection is epidemiologically associated with SSNHL, but pathogen-specific evidence for individual respiratory viruses remains predominantly case-based or serological.
				Adenovirus was isolated from a nasopharyngeal swab in a patient with sudden hearing loss, together with a fourfold rise in adenovirus-specific antibody titer [66].	4	
				An uncontrolled paired-serum series of 24 SSNHL patients identified recent influenza A or parainfluenza infection in selected cases [51].	4	
				H1N1-associated sudden hearing loss has been reported in adults and children, sometimes with PCR-confirmed infection [67,68,69].	4	
				In an uncontrolled paired-serum series of 77 patients with idiopathic sudden hearing loss, influenza B seroconversion occurred in 14 patients (approximately 18%) [18].	4	
				Influenza B seroconversion was significantly more frequent in patients with idiopathic sudden hearing loss than in controls [19].	3b	
Human immunodeficiency virus (HIV)	SSNHL may be unilateral or bilateral, although auditory disorders in HIV are heterogeneous and may also be conductive or mixed.	Confirmed HIV infection plus evaluation for opportunistic CNS/otologic infection, ototoxic drug exposure and other competing causes.	No HIV-specific treatment effect on acute SSNHL has been established. Responses to corticosteroids are inconsistent, with both hearing recovery after high-dose prednisolone and persistent hearing loss despite corticosteroid therapy reported in individual cases [146,147]. Hearing outcomes may also be influenced by opportunistic infections, CNS involvement, ototoxic drugs, and other competing etiologies [72,73,74].	Early case reports documented SSNHL as a presenting or associated manifestation of HIV/AIDS [70,71].	4	Moderate. Population-level association exists, but attribution to HIV itself is complicated by opportunistic infections, CNS disease, ototoxic drugs and other confounders.
				A population-based retrospective cohort compared 8760 patients with HIV with 43,800 controls and found an adjusted HR of 2.169 (95% CI, 1.071–4.391) for SSNHL among those aged 18–35 years [75].	2b	
				Human pathological and neurological reports demonstrate potential auditory-nerve or CNS involvement in HIV/AIDS, although opportunistic infections and other competing causes are common [72,73,74].	4 (indirect evidence)	
Lassa virus	Bilateral SNHL often occurs during convalescence, although acute and early-onset hearing loss also occur; hearing loss can be severe and persistent.	Lassa-virus RT-PCR or other virological confirmation in an appropriate systemic illness; audiometry during acute disease and convalescence.	No proven hearing-restorative therapy exists. Hearing loss may develop despite ribavirin treatment and persist for years [80].	Early hospital case series and case reports documented SNHL during or after laboratory-confirmed Lassa fever [76,80].	4	Strong. Repeated laboratory-confirmed comparative human studies and supportive animal pathology provide convergent evidence, although not all reported cases fulfill the conventional ≤72-h SSNHL definition.
				A prospective audiometric evaluation of 69 hospitalized febrile patients found SNHL in 14/49 (29%) confirmed Lassa-fever patients versus 0/20 febrile controls [79].	2b	
				A prospective study described by its authors as case–control found early-onset SNHL in 5/37 (13.5%) confirmed Lassa-fever patients and in none of the other-febrile-illness controls [81].	3b	
				Animal models demonstrated hearing loss, cochlear viral antigen, and injury to spiral-ganglion neurons and highly vascularized cochlear cells [148].	5 (indirect evidence)	
				Nonhuman-primate and mouse studies support immune-mediated neurovascular injury, including perivascular inflammation and CD4+ T-cell-dependent hearing loss [149,150].	5 (indirect evidence)	
Hepatitis B virus (HBV)	Usually reported as unilateral SSNHL, often with tinnitus; overt hepatitis symptoms may be absent.	HBV serology/HBV DNA and liver-function testing in an appropriate clinical context; MRI can support labyrinthine involvement.	Intratympanic corticosteroids may provide partial hearing improvement: in a retrospective series of 77 HBV-positive patients with unilateral SSNHL, the mean hearing gain was 15.51 dB, although 32.5% showed no improvement [151]. Evidence that HBV-directed antiviral therapy modifies hearing outcomes is currently lacking.	A case of acute exacerbation of chronic HBV infection presented with unilateral SSNHL, elevated HBV DNA, and MRI evidence of labyrinthitis [82].	4	Moderate. Population-based cohort studies support an association, but direct inner-ear HBV evidence is lacking and immune/vasculitic mechanisms may contribute.
				A retrospective matched cohort compared 170,942 patients with HBV/HCV infection with 512,826 controls; the HBV subgroup had an adjusted HR of 5.110 (95% CI, 4.495–5.809) for SSNHL [83].	2b	
				A nationwide retrospective cohort compared 33,234 HBV patients with 132,936 controls and reported an adjusted HR of 1.315 (95% CI, 1.148–1.506) for SSNHL [84].	2b	
				A cross-sectional comparative audiological study of 95 HBV-positive patients and 97 HBsAg-negative controls found higher hearing thresholds in the HBV group; temporal sequence was not established [85].	4	
				HBV-associated polyarteritis nodosa and human temporal-bone vasculitic pathology provide indirect immune-vascular support [87,88].	4 (indirect evidence)	
Enteroviruses	Reported phenotype includes sudden severe bilateral SNHL with tinnitus, sometimes accompanying CNS infection.	RT-PCR in blood/CSF or other relevant specimens; CSF testing is particularly informative when neurological disease is present.	One virologically supported case improved substantially within 5–10 days after methylprednisolone pulse therapy [89]; no controlled virus-specific treatment evidence.	A patient with sudden severe bilateral SNHL had enterovirus-positive CSF PCR and the same echovirus-4 serotype cultured from stool [89].	4	Moderate. Pathogen-specific evidence can be convincing in individual cases, but epidemiological screening results vary considerably.
				A controlled virological study detected enteroviral RNA by RT-PCR in 40% of 55 SSNHL patients and in none of the controls [90].	3b	
				A prospective screening series of 48 unilateral ISSNHL patients found enterovirus RNA in only 1 (2.08%); no non-SSNHL comparison group was included [20].	4	
West Nile virus (WNV)	Usually bilateral SNHL in neuroinvasive disease, often accompanied by encephalopathy, tremor, weakness, disequilibrium or cranial-neuropathic features; outcomes are variable.	Virological confirmation of WNV in the context of compatible neuroinvasive disease; neurological evaluation is central.	No specific therapy has been proven to improve WNV-associated hearing loss. Reported outcomes range from irreversible deafness to substantial spontaneous recovery with supportive care [91,95].	Individual cases have described severe bilateral SNHL associated with WNV meningoencephalitis or encephalitis [91,94,95,96].	4	Limited. Only a small number of case reports are currently available, and SSNHL appears to be rare.
				An uncontrolled longitudinal follow-up of WNV-infected participants documented persistent hearing abnormalities; no WNV-negative comparison group was included [97].	4 (indirect evidence)	
				A 1-year follow-up of WNV-encephalitis survivors reported decreased hearing in 7.4% of assessed survivors; no uninfected comparison group was included [98].	4 (indirect evidence)	
Dengue virus	Acute SNHL may be unilateral or bilateral and can accompany thrombocytopenia, bleeding, neurological involvement or microcirculatory disturbance; tinnitus, vertigo and aural fullness may occur.	Dengue NS1 antigen and standard virological testing in the appropriate systemic illness; audiometry.	No established hearing-specific therapy. In a prospective pilot study, dengue-associated SNHL persisted at 3-month follow-up despite standard SSNHL management, including high-dose corticosteroids in symptomatic cases [104].	Multiple case reports/series have documented unilateral or bilateral SNHL during classical or severe dengue [99,100,101,102,103].	4	Moderate. Comparative human evidence has emerged, but sample sizes remain small and not all reported auditory abnormalities satisfy strict SSNHL criteria.
				A prospective pilot study followed 10 NS1-positive dengue patients and found audiometric SNHL in three; no non-dengue comparison group was included [104].	4 (indirect evidence)	
				A prospective case–control study compared 40 NS1-positive dengue patients with 39 patients with other febrile illnesses and found hearing loss in 15.2% versus 0% (OR 34.6, 95% CI 1.97–610) [105].	3b (indirect evidence)	
Zika virus (ZIKV)	Acute or transient SNHL after a Zika-like rash illness; neural auditory-pathway abnormalities may occur even when pure-tone thresholds are normal.	RT-PCR, acute-phase IgM and dynamic PRNT titers; exclusion of dengue is important because flavivirus serological cross-reactivity limits specificity [108,109].	Complete or partial recovery within 1 month occurred in the three initially reported adults [106]; no controlled therapeutic evidence.	A three-patient case series reported hearing loss after an acute rash illness; only one case was definitively laboratory-confirmed as ZIKV, while the others had dengue serological overlap [106].	4	Limited. Evidence is restricted to small case series, and ZIKV–dengue serological cross-reactivity substantially limits causal attribution.
				Two additional case reports described auditory abnormalities after ZIKV infection, but both patients had serological evidence of both ZIKV and dengue exposure [107].	4 (indirect evidence)	
Severe acute respiratory syndrome coronavirus 2 (SARS-CoV-2)	Most reports describe acute unilateral SSNHL, often with tinnitus/vertigo and high-frequency involvement; bilateral severe loss also occurs. SSNHL may follow severe COVID-19 or occur during mild/asymptomatic infection.	SARS-CoV-2 PCR or documented recent infection; serology can provide retrospective supportive evidence. MRI may demonstrate cochlear/labyrinthine abnormalities but is nonspecific.	Most reported patients received systemic or intratympanic corticosteroids; outcomes were heterogeneous, with partial recovery common and approximately one-quarter showing little or no meaningful improvement [113,114,115,116].	Case reports and case series documented temporally associated SSNHL across severe, mild, and asymptomatic COVID-19 presentations [110,111,112,113,115,116].	4	Strong. Multiple large population-based and case–control studies consistently demonstrate an increased risk of SSNHL following SARS-CoV-2 infection, supported by human inner-ear susceptibility studies and experimental evidence of direct cochlear infection.
				A nationwide population-based cohort of 6,716,879 young adults found SSNHL incidence of 1.8 versus 0.5 per 10,000 person-months after COVID-19 versus no COVID-19 (adjusted subdistribution HR 3.52, 95% CI 3.23–3.83) [117].	2b	
				A matched Malaysian case–control study found previous COVID-19 infection in 8.6% of 187 ISSNHL cases versus 4.7% of 935 controls (adjusted OR 2.49, 95% CI 1.176–5.260) [118].	3b	
				Human inner-ear tissue and organoid experiments demonstrated SARS-CoV-2 entry-factor expression and susceptibility of inner-ear cell populations to infection [152].	5 (indirect evidence)	
				In K18-ACE2 mice, SARS-CoV-2 entered the inner ear, preferentially affected spiral-ganglion neurons, and induced molecular stress and neuronal apoptosis [153].	5 (indirect evidence)	

* Evidence levels were assigned with reference to the Oxford Centre for Evidence-Based Medicine (OCEBM) Levels of Evidence (March 2009), Therapy/Prevention, Aetiology/Harm domain. Levels used in this table are: 2b, individual cohort study; 2c, outcomes research or ecological study; 3b, individual case–control study; 4, case series or poor-quality cohort/case–control study; and 5, expert opinion or evidence based on physiology, bench research, or first principles. In accordance with the OCEBM definition of a poor-quality cohort, prospective or retrospective surveillance/series lacking a clearly defined non-exposed comparison group were graded Level 4 for the aetiologic question. Cross-sectional comparative studies without an established temporal sequence were also graded Level 4. The single case-crossover study was graded Level 3b as a self-matched analytical case–control design. Evidence levels followed by “(indirect evidence)” indicate supportive evidence that does not directly evaluate clinically defined SSNHL, including evidence from congenital, progressive, or otherwise non-SSNHL hearing-loss phenotypes; broader auditory abnormalities; human pathological or molecular studies not tied to acute SSNHL; and animal, organoid, or in-vitro mechanistic models. † Overall strength of association with SSNHL was categorized as Strong, Moderate, or Limited through qualitative synthesis of the highest-quality SSNHL-specific human evidence, consistency across independent studies, pathogen-specific confirmation, temporal and phenotypic concordance, direct inner-ear or histopathological evidence, and supportive experimental data. High-quality evidence for congenital, progressive, or other non-SSNHL hearing loss was considered supportive evidence only and did not independently increase the strength rating for SSNHL.

## Data Availability

No new data were created or analyzed in this study. Data sharing is not applicable to this article.
